# Noise-Adaptive Visible Light Communications Receiver for Automotive Applications: A Step Toward Self-Awareness

**DOI:** 10.3390/s20133764

**Published:** 2020-07-05

**Authors:** Alin-Mihai Căilean, Mihai Dimian, Valentin Popa

**Affiliations:** 1 Department of Computers, Electronics and Automation, Stefan cel Mare University of Suceava, 720229 Suceava, Romania; dimian@usm.ro (M.D.); valentin@eed.usv.ro (V.P.); 2 Integrated Center for Research, Development and Innovation in Advanced Materials, Nanotechnologies, and Distributed Systems for Fabrication and Control, Stefan cel Mare University of Suceava, 720229 Suceava, Romania

**Keywords:** adaptive communication, context-adaptive receiver, inter-vehicle communications, reconfigurable computing, SNR-adaptive receiver, visible light communication

## Abstract

Visible light communications are considered as a promising solution for inter-vehicle communications, which in turn can significantly enhance the traffic safety and efficiency. However, the vehicular visible light communications (VLC) channel is highly dynamic, very unpredictable, and subject to many noise sources. Enhancing VLC systems with self-aware capabilities would maximize the communication performances and efficiency, whatever the environmental conditions. Within this context, this letter proposes a novel signal to noise ratio (SNR)-adaptive visible light communication receiver architecture aimed for automotive applications. The novelty of this letter comes from an open loop signal processing technique in which the signal treatment complexity is established based on a real-time SNR analysis. So, the receiver evaluates the SNR, and based on this assessment, it reconfigures its structural design in order to ensure a proper signal treatment, while providing an optimal tradeoff between communication performances and computational resources usage. This approach based on software reconfiguration has the potential to provide the system with enhanced flexibility and enables its usage in resource sharing application. As far as we know, this approach has not been considered in vehicular VLC systems. The performances of the proposed architecture are demonstrated by simulations, which confirm the SNR-adaptive capacity and the optimized performances.

## 1. Introduction

As the demand for wireless communication technologies is growing, visible light communications (VLC) are developing as a promising wireless communication technology. In VLC, the data is modulated onto the instantaneous power of the light (380–780 nm), and so the information transfer is performed as a supplementary function, in addition to lighting. Consequently, VLC provides a 400 THz unlicensed bandwidth available in any place where there is LED lighting. The VLC performances have been confirmed in indoor applications, demonstrating that this technology is suitable for high-speed personal links [1,2,3]. However, after the initial purpose was mainly focused on providing record breaking wireless data rates, in the subsequent step, part of the efforts was focused on identifying new prospective applications. Thus, the VLC technology is envisioned for usage in a wide variety of applications such as device-to-device connections [4], internet of things applications [5], communications in underground mines [6], distance estimation [7], indoor localization [8,9], or even in intruder tracking [10].

Additionally, as LED lighting systems are on the way of being integrated in vehicle lighting systems and also in public street lighting, traffic lights, or traffic signs [11], using the VLC technology in automotive applications is only straightforward. In this area, the increased potential, the universal character, and the high performance to cost ratio make VLC a promising wireless communication technology for vehicular applications [12], particularly in scenarios in which a high number of vehicles are in the same area [13]. Consequently, vehicle-to-vehicle (V2V) [14,15,16] and infrastructure-to-vehicle (I2V/V2I) [17,18,19] VLC prototypes had been developed, providing encouraging results [12,20]. However, in order to be fully compatible with vehicular communications, VLC systems still need to enhance their performances [12]. Therefore, although remarkable progress has been achieved, the utilization of the VLC technology in automotive safety applications is very challenging as these use cases require high packet delivery (PDR) ratios, reduced latencies, and low bit error ratios (BER). Nevertheless, guaranteeing these characteristics becomes problematic as the outdoor VLC channel involves various sources of optical noise, whereas the vehicular channel involves variable communication distances, a high degree of mobility and unpredictable conditions [21,22]. Thus, current VLC prototypes are not capable to simultaneously address all the problems generated by the utilization of VLC in automotive applications. So, certain vehicular VLC systems are capable to deliver enhanced resilience to noise [17,19], some are able to offer high data rates [23], whereas others are able to comply with mobile conditions [24]. However, addressing all these aspects simultaneously is still a challenge, whereas an adequate response to this challenge might determine the moment when the technology is ready for commercial deployment.

**Figure 1:**
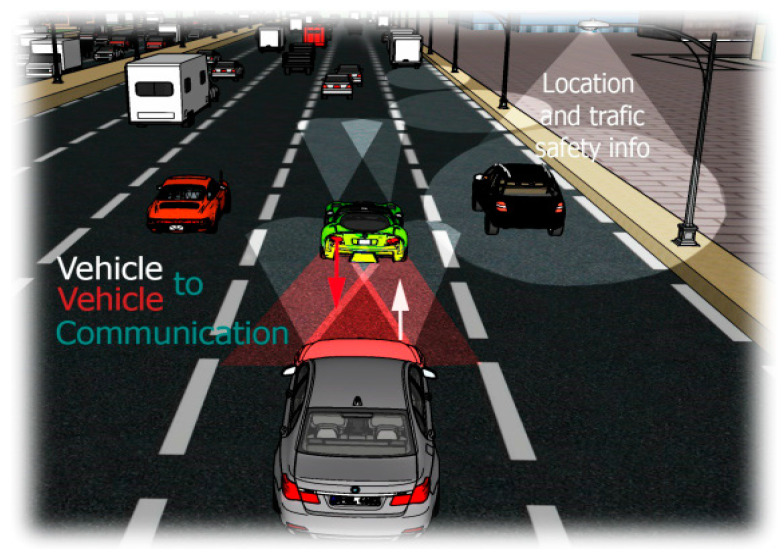
Visible light communications (VLC) usage scenario in vehicular applications.

Figure 1 illustrates a perspective regarding the utilization of the VLC technology in vehicular applications highlighting vehicle-to-vehicle and infrastructure-to-vehicle use cases. In this scenario, neighboring vehicles can exchange safety relevant information using their lighting systems, whereas traffic infrastructures that have lighting functions contribute to road safety by providing location specific data to the approaching vehicles.

Inspired by living examples, self-awareness emerges as a significant research topic in a diversity of disciplines, whereas its applicability is on the way of being explored in numerous application areas. Self-awareness promises to enable highly resilient, adaptive and exceptionally efficient behaviors. Thus, the performances of automotive VLC systems could be maximized by enabling an environment-adaptive character [25]. In addition to the enhanced performances of digital filters which provide a stronger tool for noisy signals filtering, digital signal processing (DSP) techniques also enable a higher degree of flexibility in usage. Therefore, such VLC systems can be simply reconfigured for different circumstances and environments [26]. So, a DSP-based system can adjust its cut-off frequency simply by using a different set of coefficients, whereas in noisy situations, the filtering and in turn the signal to noise ratio (SNR) can be improved by increasing the filter’s order.

In this context, this letter proposes an enhanced VLC receiver architecture based on DSP techniques. The proposed concept is capable to evaluate and determine the noise intensity and then, based on this assessment, to auto-adjust its configuration in order to provide a low BER even in low SNR conditions. On the other hand, in high SNR conditions, the VLC receiver is able to use less computational resources. The VLC receiver auto-adaptive character is enabled with the help of a SNR estimation unit, of an adaptive digital filtering mechanism and of an adaptive signal reconstruction block. The suitability of the proposed architecture is confirmed by simulations. As far as we know, this is one of the few concepts of SNR-adaptive VLC receivers designed for outdoor low data rate uses, as the automotive applications. The rest of this letter is organized as follows. Section 2 defines some of the problems related to the vehicular VLC channel, Section 3 provides the description of the proposed VLC architecture, Section 4 presents the simulation results, Section 5 debates the aspects related to the novelty the proposed concept, whereas Section 6 delivers the conclusions of this letter.

## 2. Issues Associated to the Vehicular Visible Light Communications (VLC) Channel

The mandatory line of sight (LoS) between VLC emitter and VLC receiver is a specific condition required for a VLC system to function. Basically, the modulated light must reach the receiver photosensitive element, which is usually a reversed bias positive intrinsic negative (PIN) photodiode connected in a transimpedance circuit (or a camera-based receiver, as in [24]). The photodiode will generate an electrical current dependent of the incident light which will be further processed and then it will be used for the data decoding.

The vehicular VLC channel is degraded by numerous noise sources and by abundant perturbing factors. In comparison to the indoor links, the communication distance is significantly greater and therefore, the optical irradiance of the received signals is significantly lower (few tens of nW/cm^2^), considerably affecting the SNR. Furthermore, as the vehicles are in continuous movement, the emitter–receiver distances are always changing, modifying the SNR level and making the channel highly dynamic and very unpredictable. The unpredictability of the VLC channel is also increased by the weather factors. Thus, the snowfall or the dust can obstruct light passage, affecting the intensity of the received signal, whereas the rain or the fog absorb, scatter, and reflect the incident light [12,27]. However, the strongest disturbing factor is represented by the sunlight incident on the receiver’s photoelement [12,21]. Even if the introduced DC component can be filtered, the background light introduces a strong shot noise component, which is the main source of noise in daytime conditions. In the absence of a perturbing parasitic light source, the preamplifier thermal noise is dominant. Since both shot noise and thermal noise are signal-independent and Gaussian, the noise affecting the VLC channel is modeled as additive white gaussian noise (AWGN) [28].

Recent work in the development of enhanced transimpedance solutions and improved VLC receivers have showed that there are encouraging perspectives concerning the usage of the VLC technology even in direct sunlight exposure [17,19] or in adverse weather conditions [27,29]. In such cases, an adequate design of the VLC receiver can prevent transimpedance circuit saturation even in strong sunlight exposure. Nevertheless, when such conditions are fulfilled, the amplitude of the data signal and in turn the SNR are significantly affected. So, although part of the problem was solved, this points out that more work is required on developing improved signal processing techniques that are able to deal with low SNR signals.

## 3. Considerations on the Signal to Noise Ratio (SNR)-Adaptive VLC Architecture

Since the vehicular VLC channel is characterized as highly unpredictable, extremely dynamic, and particularly noisy, an essential issue in this domain is the design of a suitable VLC system able to support these circumstances. Hence, the VLC receiver must be able to process the low-amplitude signals and to reduce disturbances caused by environment disorders, with the purpose to provide a reliable link. As the external conditions cannot be controlled, current VLC prototypes are not fully reliable, or are designed to cope with the worst-case scenario and still maintain the active communication. Yet, it is clear that in normal working situations, the upper-mentioned approach affects the system performances. As analytically demonstrated in [25], the global performances of a VLC prototype could be considerably improved by developing a design that is capable to analyze the perturbing factors and to self-adjust its settings in order to improve the efficiency for each specific case. Thus, the solution this letter proposes opens a new paradigm concerning the development of automotive VLC systems.

Considering the dynamics and the unpredictability of the automotive VLC channel and the benefits that could be provided by an environment-adaptive capability [25], this section introduces such a VLC receiver architecture. The proposed VLC receiver is capable to evaluate the SNR of the received data signal and based on this estimation, to reconfigure its signal processing plan, with the purpose of ensuring an optimally-balanced signal treatment.

### 3.1. Discussions on the SNR-Adaptive Receiver

**Figure 2:**
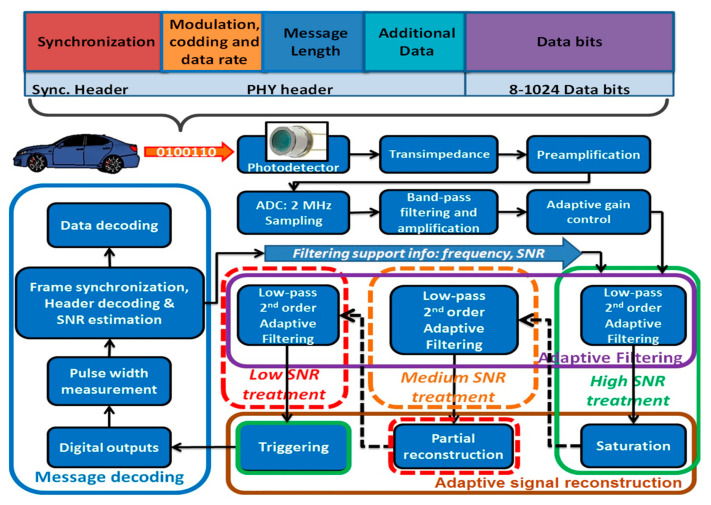
Architecture of the proposed VLC receiver.

The proposed VLC receiver is illustrated in Figure 2. Bearing in mind the digital filters enhanced filtering performances and also their superior flexibility, the signal reconstruction is mainly DSP-based. The design is developed based on a standard VLC receiver architecture [26], which was further enhanced with SNR estimation and structural design adaptation capabilities. So, this section will emphasize the aspects regarding the SNR estimation, the reconfigurable filtering, and the reconfigurable signal reconstruction, whereas other aspects regarding the architecture are considered as standard approach in the design of a VLC receiver or in DSP techniques and require no additional information.

The proposed VLC receiver concept is based on a PIN photodiode photosensitive element, which for improved resilience to noise and extended dynamic range can be implemented based on a logarithmic configuration [19]. As the signal generated by the transimpedance circuit can have amplitude levels that go down to few mV, it is preamplified in order to ensure an optimal analog to digital conversion. Next, the signal is digitalized using an analog to digital converter (ADC). At this level, the ADC sampling rate and the ADC resolution are important factors that determine the performances of the system. So, the sampling frequency will significantly influence the filtering process and the signal processing quality, while determining the computational power requirement. So, the ADC sampling frequency should be established based on a tradeoff between performances and available computational resources. Next, the signal passes through a wideband bandpass filter and it is further amplified. The bandpass filter eliminates the DC component generated by the sunlight, mitigates the effect of low frequency signals introduced by artificial light sources and also eliminates part of the high frequency noise (mostly shot noise and thermal noise). In case the system uses adaptive modulation frequency as in [26] or in [29], the band-pass filter should take this aspect into consideration by adjusting the filter’s bandpass accordingly. As the input signal can have variable intensity due to the variable emitter-receiver distance, an adaptive gain control (AGC) circuit is also integrated, providing an output signal having constant amplitude. The AGC is based on a closed-loop configuration and determines the amplitude of the output based on the average amplitude of more pulses, while also considering the minimum and the maximum values. From this point on, the VLC receiver has a basic operating mode (green block in Figure 2) involving a 2nd order Butterworth filter followed by a trigger providing the digital outputs. This operating mode is sufficient for the low priority data and can ensure a decent BER in high SNR conditions. As the SNR decreases (i.e., emitter–receiver distance increases or the incident parasitic light becomes stronger) two additional operating modes (orange block for medium SNR along with red blocks for low SNR signals) are available. Each of these operating modes adds another 2nd order Butterworth filter, whereas the low SNR mode (red block) also uses an enhanced partial signal reconstruction unit. The 2nd order filters have been selected as a fair compromise between performances and required computational power. 

In order to switch between these three working modes, the VLC design determines the channel conditions based on the received signal, by using a simple but accurate and efficient SNR determination technique. As the VLC information is modulated by switching the LEDs *On* and *Off*, these two statuses can be recognized and so, the SNR can be established. The signals corresponding for the two states are given by Equations (1) and (2).

*U_ON_* = (*R*·*P_S_* + *R*·*P_Bg_*)·*R_gain_* (1)

*U_OFF_* = (*R*·*P_Bg_*)·*R_gain_* (2)

where *R*, is the responsivity of the detector (A/W), *P_S_* is the power of the detected signal, *P_Bg_* is the power of the incident background light, and *R_gain_* is the gain resistor.

As illustrated in Figure 2 and detailed in Section 3.2, each data frame begins with a header field which contains a synchronization preamble and several other fields that provide the receiver with information which helps it during the data decoding process. While synchronizing and decoding the header information, the receiver also performs signal and noise amplitude estimation for the pulses that are included in the synchronization header. For the noise amplitude estimation, the processing unit uses the values of the unfiltered input samples, whereas the amplitude of the signal is assessed based on the values of the filtered signal. This estimation is based on a data analysis algorithm that determines the average amplitude of the *On* and of the *Off* samples, while also considering the minimum, and the maximum as control values and ignoring the samples whose standard deviation with respect to the mean values is higher than 35%. Once the VLC receiver has an assessment of the SNR, it can then decide on the proper configuration to be used. To ensure the optimal reception of the high priority data, this decision can take into consideration the message priority as this information can be provided in the message header.

While waiting for a message to be received, the receiver adopts the simplest configuration in order to become more energy efficient. Then, once an incoming message is detected, it switches to the most complex architecture, with three filtering stages and a complex signal reconstruction algorithm. While synchronizing, the synchronization preamble is evaluated and the SNR is estimated. As the information in the header is crucial for the proper decoding of the data, this configuration is maintained during the header decoding. After the synchronization is achieved and the header data is decoded, the processing unit selects the proper configuration according to the SNR assessment. Furthermore, it should be mentioned that in addition to the SNR-adaptive character, based on the data provided in the header, the receiver also has a modulation frequency (optical clock) adaptive character, enabling its usage in future multiple input multiple output (MIMO) applications.

As one could observe, the proposed implementation is based on an open loop configuration. Another approach would have been based on a closed-loop system with adaptive modulation and coding (AMC) and/or forward error correction (FEC). Such an approach can surely provide high performances in terms of BER and stability, as the system’s parameters can be adjusted based on a BER feedback. Nevertheless, we chose to implement an open loop system mainly due to its faster response to possible perturbations which translate into SNR alterations. Thus, as the vehicular VLC channel is highly unpredictable, in case of sudden modifications of the SNR, a closed-loop system would be affected by such modifications. So, a closed-loop system would most likely be affected by bit errors or even packet losses until a feedback is received and actions are made. Then again, the open loop approach that was followed here enables a faster response to such changes being highly suitable for unpredictable environments in which maintaining a certain BER and a high PDR is vital. On the downside, this approach has as a possible disadvantage due to the fact that in order to be highly effective, such a system requires a calibration which is more complex compared to the closed-loop approach. Nevertheless, if the calibration is accurately made, higher packet delivery ratio can be obtained.

### 3.2. Discussions on the Data Frame Structure

The structure of the data frames is provided in Figure 2. Each frame starts with a synchronization header consisting of a series of 1s and 0 s that points the beginning of the message and alerts the receiver that a new packet is being received. In order to enhance the assessment of the useful signal (for the SNR estimation), the synchronization header contains several series of 1 s (2–5 bits) separated by 0 s. Moreover, the initial longer series of 1 s is also a “wake-up call” that informs the receiver to switch on the header receiving configuration. To improve the accuracy and to optimally select the proper signal processing configuration, the SNR is determined by averaging the SNR of the header fields. However, in order to maintain the overheads as low as possible, the SNR determination uses no additional channel estimation sequence, contributing to a higher throughput. Next, there is the PHY header which contains information regarding the modulation, coding technique, data rate, message length, priority, and other optional information (e.g., dimming, etc.). The third field of the frame is the data field which has a flexible length of up to 1024 bits, suitable for vehicular communication applications [30]. 

As specified in the IEEE 802.15.7 standard for optical communication using visible light [31], the communication uses on-off keying (OOK) modulation, Manchester coding and a 200 kHz optical clock. However, unlike the standard frame which mentions the possible utilization of Reed Solomon (RS) and convolutional codes (CC), in this case no FEC codes were included, as this work is focused on determining the raw BER. Furthermore, the effect of FEC usage according to the IEEE 802.15.7 specifications is already established by the existing literature. 

## 4. Simulation Results and Discussions

**Figure 3:**
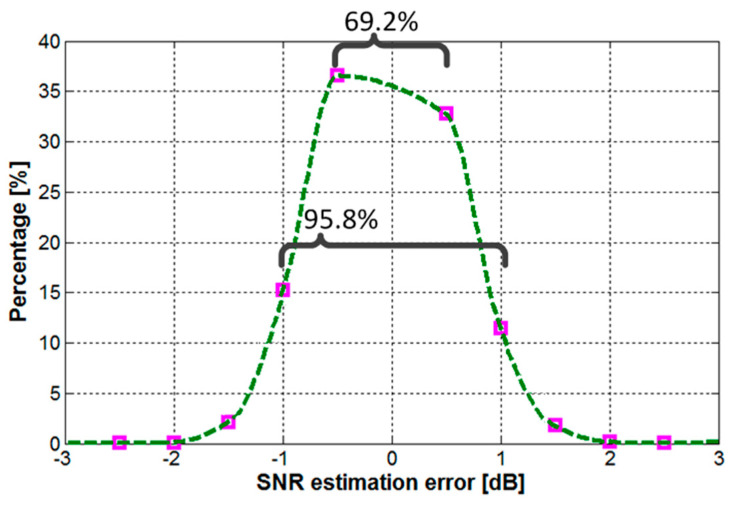
Distribution of the signal to noise ratio (SNR) assessment error at variable SNR levels between 7–15 dB.

The next section presents the simulation results confirming the performances of the proposed SNR-adaptive VLC receiver architecture. The parameters used for these simulations are summarized in Table 1. As the reconfigurable computing process is based on the SNR estimation, the first simulations were intended to evaluate the accuracy of the SNR assessment. The results of SNR estimation error for 10^4^ messages data sets are illustrated in Figure 3. One can see that in 96% of the cases, the estimation error is below 1 dB, whereas in 70%, the estimation error is below 0.5 dB, confirming the validity of the SNR approximation. The percentage estimation error has an asymmetric Gaussian distribution, slighted orientated toward the negative direction. This is because the SNR estimation algorithm was implemented with the intention to slightly diminish the estimated SNR, in order to reduce the chances that a message is treated using a suboptimal signal processing configuration. Even so, one can see that there are a limited number of messages for which the SNR estimation block considers that the SNR is higher than its real value.

**Figure 4:**
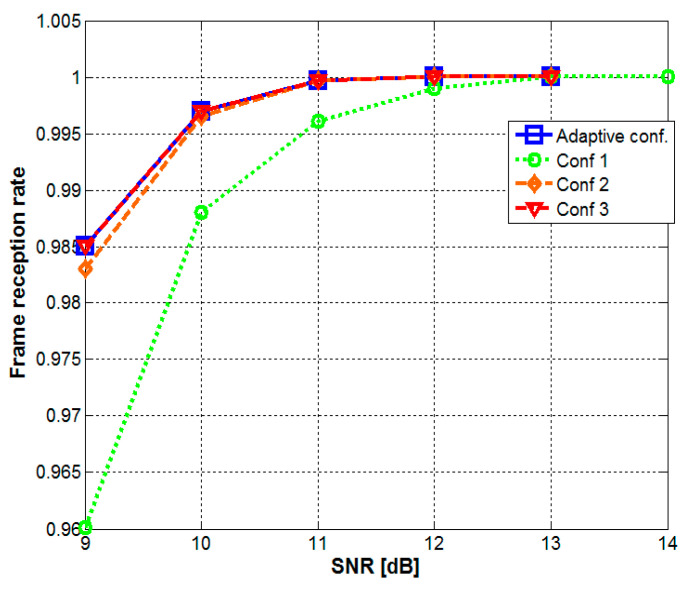
Frame delivery ratio for non-adaptive and SNR-adaptive configurations.

**Figure 5:**
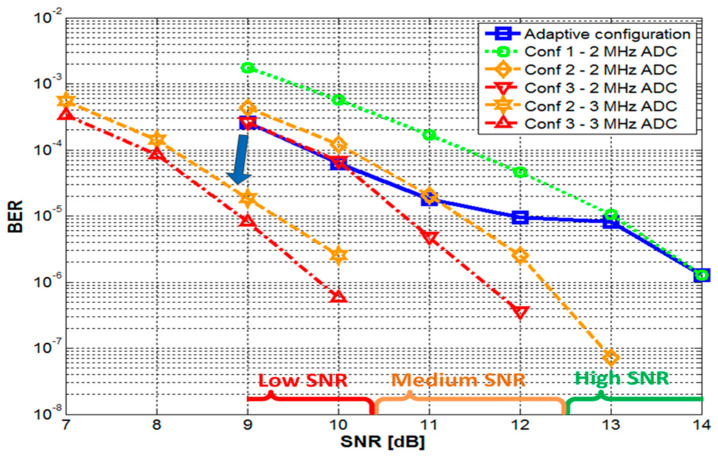
Bit error ratio for non-adaptive and SNR-adaptive configurations. Besides increasing the complexity of the signal processing algorithm, the bit error ratios (BER) can be further enhanced by increasing the sampling frequency.

After the validation of the SNR-estimation algorithm, the next simulations were aimed to investigate the performances of the three individual configurations (with no adaptive character) and to establish the SNR thresholds separating them when they are used in the SNR-adaptive configuration. Thus, assuming that a specific application requires a BER lower than 10^−4^ (e.g., a BER < 10^−3^ is required for the proper transmission of voice messages), but it has no extra benefits if the BER is lower than 10^−5^, a SNR bellow 10.5 dB was considered low (requires intensive signal treatment), whereas if it is above 12.5 dB, it is considered high (requires minimum signal treatment). With the thresholds established, the SNR-adaptive configuration was tested, in order to determine the frame reception rate and the BER. The simulations results for the frame reception rate and for the BER are presented in Figure 4, respectively in Figure 5. Due to the large data set used in these simulations, the confidence level of the results is of 99%. The results confirm the SNR-adaptive character and the optimally balanced behavior.

One can see that according to these simulations, the complex signal treatment applied to the message header maximizes the message reception rate. Regarding the BER, it can be seen that it is strongly affected by the SNR decrease. However, the receiver SNR-adaptive character reacts to the SNR deterioration and it is capable to maintain the BER within the specified limits. Still, if the SNR continues to depreciate, the receiver could react by increasing the sampling frequency (Figure 5). Thus, as more samples are used in the filtering process the performances of the filter are enhanced and accordingly, the BER results improve. Alternatively, the BER can be improved by using FEC codes [31], or by using a lower modulation frequency [26].

Most important, the results demonstrate that the performances of an automotive VLC receiver can be maximized by enabling a self-aware character [32,33] with auto-adaptive characteristics [25,34], and based on reconfigurable computing [35]. Such VLC receiver architecture is able to sustain the communication even in unfriendly conditions and enables a decent BER, while optimally harmonizing computation resource exploitation. This method is projected for utilization in vehicle embedded DSP systems based on multi-core data processing. So, the elementary working plan is handled by a VLC dedicated core, whereas when more resources are necessary, an extra core can be (re)allocated. So, when not used for the VLC signal processing, the additional core(s) can be put in standby or it can be used by other applications (resource sharing). This method brings significant resource efficiency gains while maintaining the specified performances [35]. So, it should be mentioned that as digital filters are highly complex in terms of mathematical operations, each optional signal treatment block disabling decreases the number of operations by approximately 30%.

Unlike existing VLC systems that use fixed-function hardware and/or DSP cores, this new design is more flexible, being suitable for future improvements of the operational plan or to face eventual updates in the standard. Consequently, this letter aimed to demonstrate the viability of the solution applied in automotive VLC applications rather than establishing a new design.

## 5. Concluding Remarks Concerning the proposed SNR-Adaptive Architecture, Discussions on the Benefits of the Concept, and Future Perspectives

As mentioned in Section 2 of this letter, and detailed in other works [12,19,20,21,31], there are numerous issues associated to the usage of the VLC technology in automotive applications. So, these applications are subject to numerous negative effects introduced by the environmental effects. Thus, strong sunlight or other sources of artificial light add up onto the useful data signal and tend to saturate the transimpedance circuit and significantly depreciate the SNR. Moreover, weather phenomena such as snowfall, rain or fog influence the light passage by means of light passage obstruction, reflection and refraction, again affecting the SNR [27]. The SNR is also influenced by distance modifications and spatial changes. As the distance is increasing the received optical irradiance is decreasing proportionally to the distance’s square. So, distance and spatial changes lead to significant modifications of the SNR, influencing the signal processing efficiency and accuracy. The effect of vehicle movement on the communication performances has been analyzed by simulation means in [26], showing that an adequate AGC unit can compensate vehicle movement by adjusting the receiver gain in accordance with the amplitude of the detected signal. So, in the end, all the perturbing factors from parasitic light source and adverse weather conditions to vehicle movement and spatial changes eventually affect the SNR, pointing out that a SNR-adaptive system has the potential to provide numerous benefits. In such a case, the communication limit will be established by the sensitivity of the transimpedance circuit and the ability of the signal processing plan to cope with low SNR signals. From this point on, the BER performances can be further improved by the usage of FEC algorithms. In outdoor VLC applications, the IEEE 802.15.7 standard specifies the usage of Reed Solomon and Convolutional codes to compensate the problematic outdoor VLC channel. In such cases, the maximum 100 kb/s data rate achieved for OOK modulation and Manchester coding is sacrificed in order to enhance the link resilience. Thus, the usage of FEC codes decreases the data rates to 73.3, 48.89, 24.44, respectively, to 11.67 kb/s, while providing a SNR improvement that goes up to 3 dB [31,36]. As the effect of FEC is already know by the research community, this aspect has not been further detailed in this letter.

Another aspect that worth to be mentioned is related to the interferences caused by other cars (i.e., other VLC emitters). VLC is a direct line of sight technology, which means that the VLC receiver must be orientated toward the VLC emitter in order to receive the incoming signal. Vehicular VLC applications are generally considered multi-path free, whereas the nature of light and of the VLC technology provides natural spatial isolation between neighboring communication channels. So, an on-vehicle VLC receiver is typically within the LoS of the vehicle orientated toward it. Nevertheless, if it is to think at particular situations, one can imagine a scenario in which a vehicle running on a three-lane highway has three vehicles in front, whereas one or two road side unit VLC transmitters (i.e., traffic signalization panels) are also there. In VLC, the interference between adjacent communication links has been widely addressed in indoor applications where multiple emitters and multiple users commonly share the same location. In such applications, this problem is addressed with the help of different types of multiplexing protocols (like frequency division multiplexing (FDM), time division multiplexing (TDM), and their derivatives). An analysis of these techniques together with an experimental demonstration in which up to 20 users are managed while sharing a throughput of up to 162.5 Mb/s in an office setup is provided in [37]. In vehicular applications, the problem will probably be addressed in a similar manner. Based on FDM protocols different types of messages can be transmitted on different frequencies (for example high priority messages are transited on a prioritized communication frequency). A different approach would be to use TDM protocols such as time division multiple access (TDMA) with collision avoidance in which the message is broadcasted in a randomly generated time slot. So, as the vehicular VLC channel will be in use only by few users, it will be very unlikely that two VLC emitters will transmit data simultaneously. Such multiplexing protocols have been successfully used in RF-based communication and are also in use for 5.9 GHz dedicated short-range communications (DSRC) for vehicular environments. In this case, the protocols are considered safe even when the number of vehicles that share the same communication medium is significantly greater than in VLC applications.

In this context, this letter provided the basis concerning the adaptation of the signal processing techniques to SNR, providing the VLC receiver with enhanced flexibility, improved resilience to noise and better efficiency in terms of resource utilization. Thus, the purpose of this letter is not to provide a comprehensive VLC architecture that addresses every issue associated to vehicular VLC applications, but to make a new step forward toward a self-aware automotive VLC system. At this point the SNR-adaptive VLC receiver concept presented in this letter is at simulation level. Nevertheless, based on the promising results, future work should be focused toward its hardware implementation. So, the development of environment-adaptive and context-adaptive features seems to be the most promising approach toward the deployment of the technology in real automotive applications [12,25]. In recent years, several steps in this direction have been achieved, from hardware prototypes that are able to work in totally unfriendly environment conditions [14,19,29] to enhanced systems based on the AMC concept. In this context, the future development of the SNR-adaptive VLC systems provides encouraging perspective toward the development of high-performance self-aware VLC prototypes capable to adapt to the high complexity of the vehicular communication channel.

## 6. Conclusions

This letter has introduced a novel class of VLC receivers designed for vehicular communications applications. The SNR-adaptive VLC receiver evaluates the SNR of the received signal using a simple and efficient technique, and based on this assessment it decides on the optimal signal processing scheme to be used. This type of signal processing already takes into consideration the transition trend toward self-aware systems, reconfigurable computing, and multi-core data processing units, preparing the grounds for this evolution. Simulation results confirm that such a design is able to provide robust performances in a resource-efficient manner. Thus, this new design can be considered an intermediate step toward completely self-aware environment-adaptive VLC automotive systems.

## Figures and Tables

**Table 1:** Summary of the simulation parameters.

Parameter	Feature/Value
Modulation	OOK
Coding	Manchester
Data rate	100 kb/s
Communication	Asynchronous
Header length	41 bits
Data message length	400 bits
